# Editorial: Emotions and entrepreneurship

**DOI:** 10.3389/fpsyg.2023.1255607

**Published:** 2023-08-03

**Authors:** Thierry Volery, Katia Richomme-Huet, Virginie Vial

**Affiliations:** ^1^Zurich University of Applied Sciences, Winterthur, Switzerland; ^2^Kedge Business School, Talence, France

**Keywords:** entrepreneurship, business launch, emotion, passion, affect, startup, wellbeing

Entrepreneurship is inherently an emotional process: launching and growing a business venture is often compared to a roller-coaster journey with plenty of uncertainty, risk, long working hours, and stress. It is therefore somewhat surprising that entrepreneurial emotion was largely ignored in the literature until the early 2000s. In a pioneer paper, Goss (2005) drew of Schumpeter's work to propose a theory of entrepreneurial behavior that emphasizes social interaction and emotion. Emotional energy of pride and shame are major drivers in entrepreneurship: in their continual endeavor to innovate, entrepreneurs tend to feel exhilarated and proud. But to be in a situation that demands constant innovation is, for most of them, a source of emotional distress–a feeling of constant struggle against the world.

Today, entrepreneurial emotion is broadly defined as “the affect, emotions, moods, and/or feelings that are antecedent to, concurrent with, and/or a consequence of the entrepreneurial process” (Cardon et al., 2012, p. 1). A major stream of research has focused on entrepreneurial passion (Cardon et al., 2005; Warnick et al., 2018; Obschonka et al., 2019). Passion can foster the capacity to develop new ideas that are critical to the discovery and exploitation of promising opportunities. In parallel, evidence also shows that the role of joy and fear influences the evaluation and exploitation of opportunities (Grichnik et al., 2010). Various sources of affect–social, related to object properties, cathected, event-related, self-appraisal and future appraisal, also influence opportunity identification and development (Vial and Richomme-Huet, 2021).

Over the past decade, several studies have emphasized different aspects of positive and negative emotions in entrepreneurship, the role they play in the entrepreneur's motivation, drive, creativity, success and, less frequently, failures. However, the literature has given more consideration to the consequences of affect than to its antecedents (Delgado García et al., 2015). In their recent review on entrepreneurial emotion, Javadian et al. (2022) came to a similar conclusion. They find that scant research has been conducted on the precursors of emotions (both feelings and individual emotions). Anticipated emotions deserve more attention, and the emotional influence of stakeholders on entrepreneurs is still neglected. The entrepreneurship literature also lacks research on negative affect and emotional competencies.

In the call for papers for this Research Topic, we invited scholars to investigate the role of emotions, feelings and affect in shaping the entrepreneur's mindset, motivation, attitude, personality, identity, or experience leading to the entrepreneurship process, and in interaction with other characteristics such as socio-economic background, education, gender, race, age, or personal experience. We received a total of 18 manuscripts. During the review process, 13 manuscripts were rejected, which resulted in five papers being published in this Research Topic.

Two papers contribute to the entrepreneurial passion stream. Fu et al. draw on Vallerand's dual model of passion to study the evolution of two types of entrepreneurial passion in women entrepreneurs: the harmonious entrepreneurial passion and the obsessive entrepreneurial passion. In addition, the authors provide an insight into the process by which entrepreneurial effort and the fear of failure influence entrepreneurial passion over time. Xiao and Fu investigate the impact of entrepreneurial passion across a range of life domains, while taking into account the positive emotion spillovers and work-life identity transitions. They consider entrepreneurial passion as a type of psychological resource and explore whether its positive role can spill over into family and community domains via boundary-crossing mechanisms.

The other papers focus on the relation of entrepreneurial passion with different dimensions of psychological traits and wellbeing. Drnovšek and Slavec Gomezel examine the mechanisms by which perceived stress influences entrepreneurial success. Given the difficulties that business owners face on a day-to-day basis, this study highlights the important role of positive emotions in helping business owners persevere in the face of difficulties and explores whether positive emotions moderate the relationship between perceived stress and business owner wellbeing. Liu et al. shed some light on the role of organizational resilience for the thriving and survival of enterprises during the COVID-19 pandemic. Drawing on upper echelons' theory and personality psychology, they present a model in which entrepreneurial mindfulness and entrepreneurial resilience shape organizational resilience in small businesses. Finally, in their data report, Anwar et al. provide an insight into a rich dataset built along seven cognitive, psychological, and contextual variables which track the phenomenon of entrepreneurial intention among the students of three different Indian universities. Their dataset also provides a multidimensional measurement of fear of failure.

## Author contributions

TV: Conceptualization, Writing—original draft, Writing—review and editing. KR-H: Writing—original draft, Writing—review and editing. VV: Writing—original draft, Writing—review and editing.
